# The Regulation of Exosome-Derived miRNA on Heterogeneity of Macrophages in Atherosclerotic Plaques

**DOI:** 10.3389/fimmu.2020.02175

**Published:** 2020-09-10

**Authors:** Ximing Li, Xinyong He, Junyan Wang, Dan Wang, Peiwei Cong, Aisong Zhu, Wenna Chen

**Affiliations:** ^1^Key Laboratory of Ministry of Education for TCM Viscera-State Theory and Applications, Liaoning University of Traditional Chinese Medicine, Shenyang, China; ^2^The First Medical College, Guangzhou University of Chinese Medicine, Guangzhou, China; ^3^Department of Medical Science of Laboratory, Liaoning University of Traditional Chinese Medicine, Shenyang, China; ^4^Basic Medical College, Zhejiang Chinese Medical University, Hangzhou, China

**Keywords:** exosomes, macrophages, miRNA, atherosclerosis, networks

## Abstract

Exosomes are nanosized vesicles secreted by most cells, which can deliver a variety of functional lipids, proteins, and RNAs into the target cells to participate in complex intercellular communications. Cells respond to certain physical, chemical, and biological stimuli by releasing exosomes. Exosomes are rich in small molecules of RNA, including miRNAs and mRNAs, which have been demonstrated to have certain functions in recipient cells. Recent studies on single-cell RNA sequences have revealed the transcription and the heterogeneity of macrophages in Ldlr-/-mice fed with a high-fat diet. Five macrophage populations were found in the atherosclerotic plaques. It is worth noting that these subset populations of macrophages seem to be endowed with different functions in lipid metabolism and catabolism. A total of 100 differentially expressed mRNAs were selected for these subset populations. Importantly, these macrophage populations were also present in human advanced atherosclerosis. To clarify the specific functions and the regulatory mechanism of these macrophage populations, we extracted exosome RNAs from the plasma of patients with chronic coronary artery disease (CAD) and performed RNA sequencing analysis. Compared with the healthy control, a total of 14 miRNAs were significantly expressed in these patients. A total of 5,248 potential mRNAs were predicted by the bioinformatics platform. Next, we determined the outcome of the intersection of these predicted mRNAs with 100 mRNAs expressed in the above-mentioned five macrophage populations. Based on the screening of miRNA–mRNA pairs, a co-expression network was drawn to find out the key RNAs. Three down-regulated miRNAs and five up-regulated mRNAs were selected for validation by real-time RT-PCR. The results showed that the expression of miR-4498 in plasma exosomes was lower than that in the healthy control, and the expressions of Ctss, Ccr2 and Trem2 mRNA in peripheral blood mononuclear cells isolated from CAD patients were higher. In order to clarify the regulatory mechanism, we established a co-culture system *in vitro*. Studies have shown that the uptake of exosomes from CAD patients can up-regulate the expression of Ctss, Trem2, and Ccr2 mRNA in THP-1 cells induced by lipopolysaccharide. Our findings revealed a unique relationship between the transcriptional signature and the phenotypic heterogeneity of macrophage in the atherosclerotic microenvironment.

## Introduction

Exosomes are nanosized vesicles secreted by most cells, which can deliver a variety of functional lipids, proteins, and RNAs into the target cells to participate in complex intercellular communications. Cells respond to a series of physical, chemical, and biological stimuli (such as inflammation, oxidative stress, and hypoxia) by releasing exosomes. Exosomes are rich in small molecules of RNA, including miRNAs and mRNAs, which have been demonstrated to have certain functions in recipient cells. The exosomes of RNAs can change the gene expression in those cells (1).

Many studies have shown that the release of exosomes following ischemic injuries affects not only the cardiovascular cells but also the cells in the microenvironment, thereby modulating the repair process (2). Exosomes could regulate the differentiation, proliferation, and remodeling of cardiomyocytes, fibroblasts, and inflammatory cells (3–5). As a kind of lipid mediators, exosomes can deliver lipids and lipolytic enzymes, and their biosynthesis requires specific lipids and membrane reorganization. This study aimed to provide a comprehensive insight into chronic inflammatory diseases to further understand the importance of exosomes in solving inflammatory response and to explore new regulatory mechanisms.

It was found that several functional phenotypes of macrophages respond to the microenvironment and play different roles in vascular inflammation and atherosclerosis. Various macrophage populations were found in atherosclerotic plaques (6). It is worth noting that these macrophage subset populations seemed to be endowed with different functions in lipid metabolism and catabolism.

To clarify the specific functions and the regulatory mechanism of these macrophage subsets and the regulatory mechanism, we set out to reveal the unique relationship between the transcriptional signature and the phenotypic heterogeneity of macrophages in the atherosclerotic microenvironment.

## Materials and Methods

### Sample Collection

Cases of patients of stable angina (SA) with significant coronary artery stenosis (> 50%) were collected (*n* = 15). The age of the patients ranged from 45 to 75 years old. No patient was under medication with corticosteroids or non-steroidal anti-inflammatory drugs, except for aspirin. No patient had concurrent inflammatory or neoplastic conditions likely to be associated with an acute-phase response. Selective coronary angiography was performed in multiple views using the standard Judkins techniques to figure out the number of coronary artery stenosis in terms of single- or multiple-vessel stenosis. The ethical approval for the study was authorized by the institutional ethical committee of Liaoning University of Traditional Chinese Medicine, and all participants provided informed consent for the experiment. The control group in this study was composed of age-matched healthy volunteers (*n* = 15) compared to patients with stable angina. The demographic and clinical data are shown in Table 1.

**TABLE 1:** Demographic and clinical characteristics of the coronary artery disease (CAD) patients and the controls.

**Variable**	**Control (*n* = 15)**	**CAD (*n* = 15)**
Age (year)	56 ± 11	55 ± 10
Sex (M/F)	10/5	9/6
Smokers, *n* (%)	5 (33)	4 (26)
Hypertension, *n* (%)	8 (53)	3 (20)
Diabetes mellitus, *n* (%)	3 (20)	0
Coronary artery stenosis (%)	61 ± 13	8 ± 5**
TC (mmol/L)	4.43 ± 0.72	5.05 ± 0.94
TG (mmol/L)	0.94 ± 0.41	1.96 ± 1.04*
LDL-C (mmol/L)	2.84 ± 0.79	3.43 ± 0.66*
HDL-C (mmol/L)	1.44 ± 0.37	1.18 ± 0.32
Apo A1 (mmol/L)	1.27 ± 0.33	0.95 ± 0.22
Apo B (mmol/L)	0.92 ± 0.32	0.87 ± 0.23
FBG (g/L)	2.91 ± 0.85	2.16 ± 0.73

TC, total cholesterol; TG, triglyceride; LDL-C, low-density lipoprotein cholesterol; HDL-C, high-density lipoprotein cholesterol; FBG, fibrinogen; Apo A1, apolipoprotein A1; Apo B, apolipoprotein B. **p < 0.01 or *p < 0.05 vs. control.

### RNA Isolation and Sequencing

Total Exo-RNAs were extracted from the plasma derived from four individuals in the coronary artery disease (CAD) group or the control group. ExoRNeasy serum/plasma Maxi kits (QIAGEN, Cat. No. 77064) were used according to the instruction. Pre-filtered (with 0.8-μm filter) plasma was used to exclude cell contamination and mixed with the same volume of XBP buffer. The exosomes were bound to an exoEasy membrane affinity spin column after centrifuging at 500 × *g* for 1 min. Then, the bound exosomes were washed with 10 ml XWP buffer by centrifuging at 500 × *g* for 1 min. A total of 700 μl QIAzol was added to the membrane. Spinning went on for 5 min at 5,000 × *g* to collect the lysate, which was transferred completely to a 2-ml tube. Then, 90 μl chloroform was added to the tube containing the lysate for 15 s, followed with centrifuging for 15 min at 12,000 × *g* at 4°C. The upper aqueous phase was transferred to a new collection tube, and two volumes of 100% ethanol were added prior to mixing thoroughly. Up to 700 μl of the sample was pipetted into a RNeasy MinElute spin column in a 2-ml collection tube and centrifuged at ≥ 8,000 × *g* for 15 s at room temperature (RT). Then, the flow-through was discarded. RWT buffer at 700 μl was added to the column and centrifuged for 15 s at ≥ 8,000 × *g*. A total of 500 μl of RPE buffer was pipetted onto the column and centrifuged for 15 s at ≥ 8,000 × *g*. The step was repeated and centrifugation was carried out for 2 min at ≥ 8,000 × *g*. Then, 14 μl of RNase-free water was added directly to the column membrane, and the mixture was centrifuged for 1 min to elute the RNA. The RNA concentration was detected by the RNA Assay Kit (Life Technologies, CA, United States).

miRNA-seq was analyzed by Genesky Biotechnologies Inc., Shanghai. The libraries for RNA-seq were performed according to the Illumina Truseq small RNA protocol and sequenced with HiSeq 3000 (Illumina Inc.). Mirdeep2 software^1^ (7) was used to compare the small RNA sequence of each sample with the miRNA precursor and the mature sequence of the corresponding species in the miRBase database^2^. The known miRNAs and their secondary structure were identified by comparison with the homologous miRNA sequences of the related species. The counting numbers of the known miRNAs in each sample were calculated. The target genes of the miRNAs were predicted by TargetScan^3^ and StarBase^4^.

### Differential Analysis on the Expression of mRNAs in Monocyte-Related Populations in AS Plaques

Clément performed single-cell RNA sequencing analysis on CD45^+^ cells extracted from the aorta of chow-fed mice and atherosclerotic Ldlr^–/–^ mice fed with a high-fat diet for 11 weeks (*n* = 10) (6). The aortas were pooled to generate the samples used for single-cell RNA sequencing. Then, a two-dimensional space through t-stochastic neighbor embedding (t-SNE) was used to identify overlapping and atherosclerosis-associated immune cell populations. Thirteen distinct aortic cell clusters were singled out. In the myeloid cell populations, five monocyte-related populations were found, including resident macrophages, monocytes, monocyte-derived dendritic cells, inflammatory macrophages, and triggered receptor expressed on myeloid cells 2 (TREM2^hi^) macrophages. Differential gene expression and gene ontology enrichment were analyzed to distinguish from these five clusters. A heatmap plot was drawn using BioJupies, a website that automatically generates RNA-seq data analysis (8), to show the differentially expressed genes characteristic of the five clusters of monocyte-related populations. In the clustering analysis, the up-regulated and the down-regulated genes were colored in red and blue, respectively. To find out the key mRNAs, we generated the diagram with Venny 2.1.0^5^ to show the number of unique and shared mRNAs experimentally identified in the five clusters.

### GO and KEGG Analysis of DEmRNAs in Aortas of Mice

We used the Gene Ontology (GO) database^6^ to perform GO analysis on the mRNAs. The *P*-value and the false discovery rate were calculated by Fisher’s exact test and multiple-comparison test, respectively (*p* < 0.05). Around 1,342 differentially expressed genes were classified in the light of the GO terms, including biological process, molecular function, and cellular component.

Pathway enrichment analyses in the aortas of mice were performed based on Kyoto Encyclopedia of Genes and Genomes (KEGG) pathway analysis by using either chi-square test or Fisher’s exact test. The pathways with more annotations in the differentially expressed genes (*p* < 0.05) were considered to be significantly enriched and used for the biological identification of each cluster (scale: log2 fold change).

### MiRNA-Seq Analysis of Plasma Exosomes From CAD Patients and Healthy Control

MiRNA-seq was analyzed, and a million counts of mapped reads for each sample were noted. The Fastq reads were checked for quality, and an interquartile range (IQR) plot, a volcano plot, and a scatter plot were drawn using BioJupies, a website which automatically generates RNA-seq data analysis (8). A principal component analysis (PCA) revealed the overall distribution of differentially expressed miRNAs. The differentially expressed miRNAs in each sample were shown in the heatmap plot.

### Constructing a miRNAs–mRNAs Interacted Network

We identified the intersection of common mRNAs among DEmRNAs and the predicted mRNAs by miRNAs. Co-expression networks were drawn by using Cytoscape 3.7.1 software based on the screening of miRNA–mRNA pairs.

Since miRNA response elements (MREs) are the mediators of mRNA–miRNA interaction, we utilized miRWalk 2.0 to identify the relationships between the 14 DEmiRNAs and the 1,342 DEmRNAs (9). Furthermore, we selected the pairs with strong correlations to construct the miRNAs–mRNAs network. The interacted networks indicated the co-expression patterns of miRNAs and mRNAs. In this network, each gene corresponds to a node, and the connection of two genes is represented by an edge which indicates a significantly negative correlation. The main miRNAs and mRNAs in the network are shown in different colors.

The highchart was set out^7^ to visualize the relationships among miRNAs, mRNA, and five monocyte-related populations. Each point consists of multiple-weighted links to other points in this chart. The key nodes were selected by dependency wheel series (≥ 5) in this network.

### Isolating Exosomes From Plasma

Exosomes were isolated from equal amounts (2 to 3 ml) of plasma (containing an anticoagulant, ethylenediaminetetraacetic acid) from control or CAD patients (*n* = 3/group) using exoEasy^®^ Serum/Plasma Maxi Kit (Cat. No. 76064, QIAGEN GmbH, Hilden, Germany). The individuals were consistent with the ones for exo-RNA sequencing. Pre-filtered (with 0.8-μm filter) plasma was used to exclude cell contamination and mixed with the same volume of XBP buffer. The exosomes were bound to an exoEasy membrane affinity spin column after centrifuging at 500 × *g* for 1 min. Then, the bound exosomes were washed with 10 ml XWP buffer by centrifuging at 500 × *g* for 1 min. Then, the exosomes were eluted with 400 μl XE buffer and were then ready for physical analysis or uptake by the recipient cells. The isolated exosomes were characterized by transmission electron microscopy, nanoparticle tracking analysis (NTA), and Western blot.

### Observation of Exosomes With Transmission Electron Microscopy

The exosomes re-suspended in phosphate-buffered saline (PBS) were loaded onto Formvar-carbon-coated electron microscopy grids that had been glow-discharged for 30 s in air. Then, the grids were immediately negatively stained using 2% phosphotungstic acid and visualized with an H-7650 transmission electron microscope (HITACHI, Tokyo, Japan) operated at 80 kV.

### Nanoparticle Tracking Analysis of Exosomes

The size distribution of exosomes was assayed using NanoSight N300 (Malvern Instruments, Malvern, United Kingdom). The samples were monitored with the use of a 640-nm laser. The frame rate used was 30 frames per second and Nanosight particle tracking software (version NTA 3.2) was used to calculate exosome concentrations and size distribution.

### Analysis of Exosome Markers by Western Blot

Protein was extracted from isolated exosomes with RIPA buffer (Sigma-Aldrich), and a total of 20 μg of protein was loaded into 10% SDS-PAGE gel for separation. Then, the protein was transferred onto a polyvinylidene fluoride membrane (Bio-Rad, CA, United States). The membrane was incubated with anti-CD9 rRabbit mAb (1:1,000, clone D8O1A, CST, #13174) or CD63 (1:1,000, Invitrogen, #10628D) overnight at 4°C after having been blocked with 5% milk. The secondary antibody was horseradish peroxidase-linked anti-rabbit IgG (1:2,000, CST, #7074) or anti-mouse IgG (1:2,000, CST, #7076). The membrane was visualized with ECL Western Blotting Detection (Tanon 5200, Shanghai, China).

### Isolating Human Peripheral Blood Mononuclear Cells

Human peripheral blood mononuclear cells (PBMCs) were isolated in Lymphoprep^TM^ and SepMate^TM^ RUO tubes (STEMCELL Technologies, United States) by using density gradient centrifugation. Then, 2.5 ml of blood was diluted with an equal amount of PBS with 2% fetal bovine serum. The blood was layered on top of 5 ml Lymphoprep^TM^, being careful to minimize the mixing of blood with Lymphoprep^TM^. The tubes were centrifuged at 800 × *g* for 20 min at room temperature with brake-off. The upper plasma layer was removed and discarded without disturbing the plasma–Lymphoprep^TM^ interface. The mononuclear cells (MNC) layer was removed and retained at the interface. The MNCs were washed once with RPMI1640 medium. The monocytes were separated from other leukocytes by adherence to plastic after being cultured in the plate for 2 h. The monocytes were collected for mRNA detection by RT-PCR (*n* = 3/group).

### Differentiating THP1 Cells Into Macrophages

The THP1 cells were differentiated into macrophages cultured in medium 1640 and supplemented with 25 nM phorbol-12-myristate-13-acetate (PMA) and 10% fetal calf serum over 48 h, followed by a recovery period of 24 h in culture in the absence of PMA (10). The THP1 cells were observed to exhibit macrophage-like characteristics, such as adherent, round, short spindle, or irregular polygons with localized protrusions.

### Uptake of Exosome-Derived miRNA by Macrophages

To examine the uptake of exosomes by THP1 cells *in vitro*, the exosomes were labeled with a PKH67 Green Fluorescent Cell Linker Mini Kit (Cat. No. MINI67, Sigma-Aldrich). Then, 1 ml of final staining volume contained final concentrations of 1 × 10^–6^ M of PKH67 and 5 μg/ml exosomes. Moreover, 0.5 μg fluorescently labeled exosomes (5 μg/ml) were then added into 1 × 10^6^ macrophages at 37°C and incubated for 12 h. Then, the labeled exosomes were captured by living cell imaging. Fluorescent cellular imaging was then performed with Cytation^TM^ 1 Cell Imaging Multi-Mode Reader (BioTek, United States) by using the GFP channel for PKH67 green-fluorescence-labeled exosomes. Cell number counting was detected in the bright field.

### RNA Isolation From Cells and Exosomes

To validate the similarities in miRNA profiles between the exosomes isolated for *in vitro* experiments and the exo-RNA extracted from plasma, we extracted RNA from exosomes isolated from plasma for quantitation of miRNAs by real-time qPCR.

Total RNA was isolated, using miRNeasy Mini Kit (cat# 217004, Qiagen GmbH, Hilden, Germany), from exosomes or cells. Briefly, the samples were added with 700 μl QIAzol lysis reagent and incubated at 25°C for 5 min. Chloroform (140 μl) was added, and the tubes were shaken vigorously for 15 s. The samples were centrifuged for 15 min at 12,000 × *g* at 4°C after incubation for 2 to 3 min. The upper aqueous phase was transferred to a new collection tube and added with 1.5 volumes of 100% ethanol with thorough mixing by pipetting. Up to 700 μl of the sample was pipetted into a RNeasy^®^ Mini column in a 2-ml collection tube and centrifuged at ≥ 8,000 × *g* for 15 s at RT, and then the flow-through was discarded. Moreover, 700 μl of RWT buffer was added to the RNeasy Mini column and centrifuged for 15 s at ≥ 8,000 × *g*, and then the flow-through was discarded. RPE buffer (500 μl) was pipetted onto the RNeasy Mini column and centrifuged for 15 s at ≥ 8,000 × g, and then the flow-through was discarded (repeated this step). The RNeasy Mini column was transferred to a new 1.5-ml collection tube, and 30–50 μl RNase-free water was pipetted directly onto the column membrane and then centrifuged for 1 min at ≥ 8,000 × *g* to elute.

### Validation of DEmiRNAs in Exosomes or DEmRNAs in PBMCs by Real-Time RT-qPCR

Total RNA was reverse-transcribed to cDNA with AMV reverse transcriptase (Takara) and a stem-loop RT primer. Real-time PCR was performed with a TaqMan PCR kit and ABI 7900 (Applied Biosystems). Exo-miRNA quantification was conducted by using the miScript II RT Kit (QIAGEN) and the miScript SYBR^®^ Green PCR Kit (QIAGEN). The RNA levels were determined by 2^–ΔΔCt^ method and normalized to β-actin and U6 for mRNAs and miRNAs, respectively (*n* = 3/group). The mature sequences of miR-4498, miR-1226-5p, and miR-320c were UGGGCUGG CAGGGCAAGUGCUG, GUGAGGGCAUGCAGGCCUGGAU GGGG, and AAAAGCUGGGUUGAGAGGGU individually. The specific primers/sequences for amplifying miRNAs and mRNA are listed in Table 2.

**TABLE 2:** Primers for reverse transcription-polymerase chain reaction.

**Genes**	**Primers**	**Accession number**
hsa-miR-4498	F: 5′-AACAATTGGGCTGGCAGGG-3′	MI0016860
	R: 5′-CAGTGCAGGGTCCGAGGT-3′	
hsa-miR-1226-5p	F: 5′-AACAAGGTGAGGGCATGCAG-3′	MI0006313
	R: 5′-GTGCAGGGTCCGAGGT-3′	
hsa-mir-320c	F: 5′-ACACAAGAAAAGCTGGGTTGAGA-3′	MI0003778
	R: 5′-CAGTGCAGGGTCCGAGGT-3′	
Ctss	F: 5′-TGGATCACCACTGGCATCTCTG-3′	NM_004079
	R: 5′-GCTCCAGGTTGTGAAGCATCA-3′	
Trem2	F: 5′-ATGATGCGGGTCTCTACCAGTG-3′	NM_018965
	R: 5′-GCATCCTCGAAGCTCTCAGACT-3′	
Ccr2	F: 5′-CAGGTGACAGAGACTCTTGGGA-3′	NM_000647
	R: 5′-GGCAATCCTACAGCCAAGAGCT-3′	

### Luciferase Reporter Assay

The sequences of Ctss 3′-UTR comprising the miR-4498 binding site were synthesized and inserted into the pMIR-REPORT^TM^ vector (Ambion) to construct a luciferase vector. Next, miRNA mimics/miR-NC (synthesized by GenePharma, Shanghai, China) and the above-mentioned luciferase vector were co-transfected into cells. The luminescence signal was detected by GloMax^®^ 20/20 Luminometer (Promega) in 48 h after co-transfection in accordance with the protocol of Dual-Glo luciferase reporter assay (Promega, WI, United States). The values of the firefly luciferase assay were normalized to the Renilla luciferase assay value from the transfected phRL-null vector (Promega).

### Inducting mRNA Expression in THP1 Cells by Exosomes From Plasma

The THP1 cells were differentiated into macrophages by culturing in 1640 medium supplemented with 25 nM PMA and 10% fetal calf serum for 48 h, followed with a recovery period of 24 h in culture in the absence of PMA. To clarify the effect of exosome-derived miRNAs on THP1 cells, we detected the mRNA expressions of Ctss and Trem2 in cells co-cultured with lipopolysaccharide (LPS), exosomes, or/and miR-4498 mimic (catalog no. 4464066, Thermo Fisher, United States)/miR-4498 inhibitor (catalog no. 4464084, Thermo Fisher, United States) in serum-free 1640 medium for 24 h with LPS induction (*n* = 3/group).

### Statistical Analysis

The statistical analyses in the two groups were performed by a two-tailed Student’s *t*-test. For multiple comparisons, the *p*-value was determined by two-way ANOVA, followed by a Bonferroni posttest. All analyses were performed with GraphPad Prism 5 software. A value of *p* < 0.05 was considered to be statistically significant. Data are shown as mean ± SD.

## Results

### Gene Expression Signature of Macrophages in the Atherosclerotic Aorta

**FIGURE 1:**
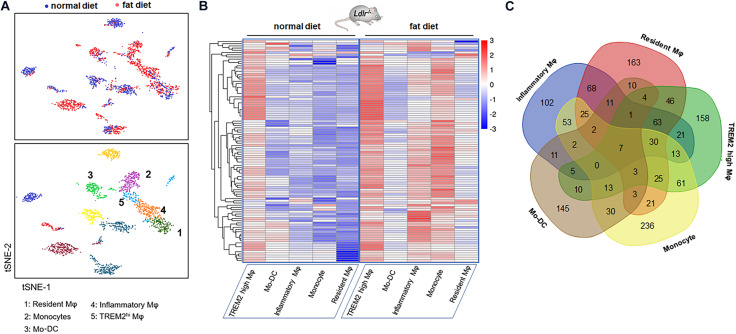
Gene expression signature of macrophages in the atherosclerotic aorta. **(A)** Five monocyte-related populations in atherosclerotic aortas from Ldlr^–/–^ mice fed with a high-fat diet by single-cell RNA-seq, followed with tSNE analysis (data from Clement et al.). **(B)** Heatmap of differentially expressed genes in five clusters. Genes were ordered by hierarchical clustering. **(C)** Venn diagram of the gene characteristics of the five clusters.

Recent research (6) revealed the transcription and the heterogeneity of macrophages in atherosclerotic aortas from Ldlr^–/–^ mice fed with a high-fat diet by single-cell RNA-seq, followed with tSNE analysis. There existed five monocyte-related populations in atherosclerotic plaques (shown in Figure 1A). Importantly, these populations were also present in human advanced atherosclerosis. Analyses on differential gene expression and gene ontology enrichment revealed specific gene expression (total of 1,342 genes) patterns that distinguished these five clusters. We analyzed the data and selected the top 20 differentially expressed genes in each cluster of populations (shown in Table 3).

**TABLE 3:** The top 20 differentially expressed genes in five monocyte-related populations.

**TREM2^high^ Mφ**	**Resident Mφ**	**Inflammatory Mφ**	**Monocytes**	**Mφ-DC**
Trem2	C1qb	C1qb	Lgals3	Syngr2
C1qb	C1qa	C1qa	Psap	Cd209a
Anxa5	Ctsc	C1qc	Msrb1	H2-Aa
Igf1	C1qc	Nfkbiz	F10	Ifi30
Cd9	Lyz2	Cd83	Cstb	H2-Ab1
Cd63	Sepp1	Cxcl2	Plin2	Gm2a
Hexb	Pf4	Egr1	Rnh1	Cd74
C1qc	Fcgrt	Socs3	Tgfbi	Flt3
Lgals3	Csf1r	Zfp36	Thbs1	H2-Eb1
Ctsb	Trf	Junb	Lilrb4a	Napsa
Ms4a7	Cd81	C5ar1	Osm	Olfm1
Prdx1	Mrc1	Csf1r	Sat1	H2afz
Cd72	Fcrls	Nfkbia	Lst1	H2-DMb1
Ctsz	Pid1	C3ar1	Fcer1g	H2-DMa
Syngr1	Serinc3	Atf3	Ctss	Atox1
Hexa	Fcgr3	Cd14	Litaf	Lsp1
Ctss	Adgre1	Cd81	Ctsb	Cbfa2t3
Cd68	Ehd4	Marcksl1	Npc2	Plbd1
Ctsd	Txnip	Fcer1g	Tyrobp	H2-DMb2
C1qa	Cfh	Adgre1	Msr1	Rogdi

The heatmap shows the hierarchical clustering of differentially expressed mRNAs in aortas from mice fed with a fat diet compared with normal control in each cluster. The heatmap of genes in each Mφ population vs. the four others as determined by single-cell differential expression analysis is shown in Figure 1B. The Venn diagram showed that there were seven co-expressed mRNAs (Tyrobp, Irf5, Pirb, Ctsh, Csf2ra, Spi1, and Ccdc109b) shared in five clusters, and there were 36 mRNAs shared in at least four clusters, such as Cst3, Ctsz, Il10rb, Cd63, Ctss, Ctsa, Lamp2, Sirpa, Apobec1, Fcer1g, Apoe, Fcgr3, Hexa, etc. (Figure 1C).

### Enrichment Analysis of GO and Pathway on Differentially Expressed Genes

**FIGURE 2:**
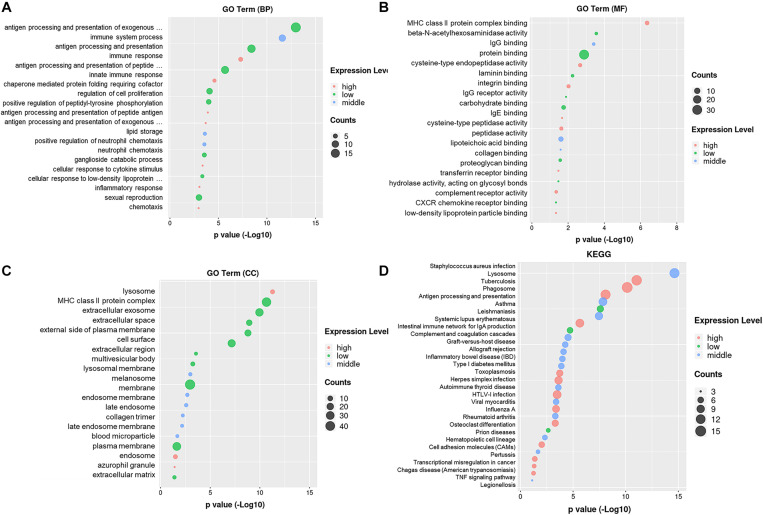
Gene ontology enrichment and KEGG analysis on DEmRNAs. **(A)** Gene ontology enrichment analysis on genes relative with the biological process. **(B)** Gene ontology enrichment analysis on genes relative with the molecular function. **(C)** Gene ontology enrichment analysis on genes relative with the cellular component. **(D)** Pathway enrichment analysis (KEGG) on genes.

The GO enrichment analysis revealed the biological process targeted by these differentially expressed genes: antigen processing and presentation, immune system process, innate immune response, regulation of cell proliferation, and positive regulation of peptidyl-tyrosine phosphorylation (Figure 2A). The gene ontology molecular function includes protein binding, MHC class II protein complex binding, peptidase activity, IgG receptor binding, lipoteichoic acid-binding, and beta-N-acetyl hexosaminidase activity (Figure 2B). The gene ontology cellular components include lysosome, MHC class II protein complex, extracellular exosome, extracellular space, cell surface, membrane, and plasma membrane (Figure 2C). The pathway enrichment analysis (KEGG) revealed lysosome, phagosome, antigen processing and presentation, TNF-signal pathway, transcriptional mis-regulation in cancer, and cell adhesion molecules. These signaling pathways and target genes may play critical roles in regulating the immune response, including the function and the phenotype of Mφ, shown in Figure 2D.

To address whether the genes enriched in macrophage subsets in murine atherosclerotic aortas could be detected in human lesions, we performed a literature screen, which revealed that the genes enriched in inflammatory macrophages (Tnf-α, Tnfsf9, Ccl3, Tlr2, Egr1, and Ccl2), Res-like macrophages (Cxcl4, Lyve1, Txnip, and Gas6), and TREM2^hi^ macrophages (Trem2 and Spp1) were detected in lesional macrophages isolated from carotid artery tissue samples from patients with high-grade carotid artery stenosis (> 70%) (6, 11–15).

### Differentially Expressed miRNAs in Plasma Exosomes From CAD Patients and Healthy Control by miRNA Sequencing

**FIGURE 3:**
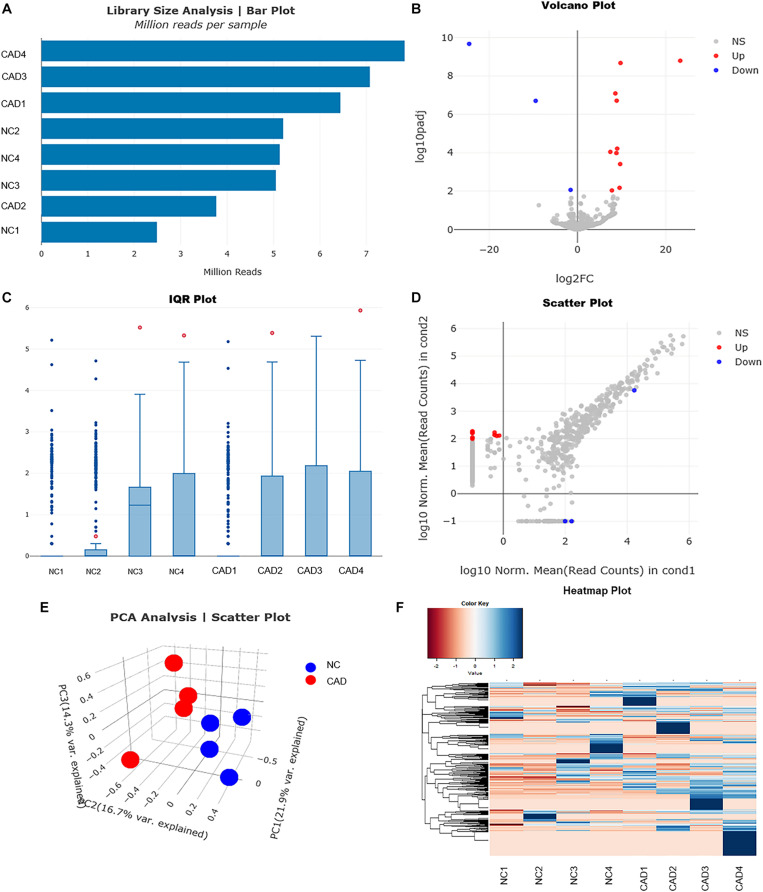
Profiles for miRNAs of plasma exosomes from healthy and coronary artery disease (CAD) individuals. **(A)** Bar plot shows the reads count distribution of each sample by library size analysis. **(B)** Volcano plot for comparing the differentially expressed genes of exosomal miRNAs in plasma from healthy and CAD individuals after analysis with TargetScan (fold change > 1.5 and *p* < 0.05). **(C)** Inter-quartile range plot shows the data dispersion of each sample. Representative box plots of two differentially expressed miRNA that were detected in EVs from either healthy or CAD plasma are shown. **(D)** Scatter plot shows the relationship of read counts and the samples between the two groups. **(E)** Principal component analysis showing the separation of samples from healthy and CAD plasma. Each point represents a sample. **(F)** Heatmaps of the top differentially expressed miRNAs from plasma exosomes of healthy and CAD patients (*n* = 4 each group) were identified in hierarchical clustering. The color that represents the specific value is provided in the legend on the upper-left side of the figure.

To explore whether the differentially expressed mRNAs were regulated by the miRNAs derived from plasma exosomes, we performed miRNA sequencing to identify differentially expressed miRNAs (DEmiRNAs) in plasma exosomes isolated from CAD patients and healthy individuals. A total of 342 known miRNAs were identified following quality control, among which 14 were relatively abundant (*p* < 0.05, > 1.5-fold change) in the plasma exosomes from CAD patients vs. those from healthy individuals, including three down-regulated (hsa-miR-320c, hsa-miR-1226-5p, and hsa-miR-4498) and 10 up-regulated miRNAs (hsa-miR-452-5p, hsa-miR-196b-5p, hsa-miR-200c-3p, etc., shown in Table 4). The bar plot shows the reads count distribution of each sample by library size analysis (Figure 3A). The volcano plot showed the differentially expressed exosomal miRNAs in plasma from healthy and CAD individuals after analysis with TargetScan (fold change > 1.5 and *p* < 0.05; shown in Figure 3B). The IQR plot showed the data dispersion of each sample (Figure 3C). The scatter plot and the PCA showed the relationship and the separation of the samples between the two groups (Figures 3D,E). The heatmap showed the top differentially expressed miRNAs from plasma exosomes of healthy and CAD patients (Figure 3F).

**TABLE 4:** DEmiRNAs in exosomes from coronary artery disease patients vs. control.

	***p*-adj**	**log2FC**	***p*-Value**
hsa-miR-452-5p	1.60E−09	23.25	6.36E−12
hsa-miR-196b-5p	2.10E−09	9.73	1.26E−11
hsa-miR-200c-3p	3.89E−04	9.66	7.75E−06
hsa-miR-15b-3p	6.72E−03	9.52	1.47E−04
hsa-miR-542-3p	6.11E−05	8.98	8.52E−07
hsa-miR-1304-5p	1.94E−07	8.87	1.93E−09
hsa-miR-29c-5p	1.04E−04	8.81	1.86E−06
hsa-miR-15b-5p	8.08E−08	8.57	6.44E−10
hsa-let-7f-1-3p	9.21E−03	7.78	2.39E−04
hsa-miR-433-3p	3.45E−05	7.57	3.02E−05
hsa-miR-221-5p	9.00E−05	7.43	1.43E−06
hsa-miR-320c	0.00863	–1.57	0.000206
hsa-miR-1226-5p	1.98E−07	–9.47	2.36E−09
hsa-miR-4498	2.12E−10	–24.53	4.23E−13

### Identification of miRNA–Target mRNAs

The miRNAs regulate the expression of specific genes by hybridization with mRNAs through MREs, thereby promoting their degradation and inhibiting their translation. To study the possible functional roles of the differentially expressed miRNAs, their potential mRNA targets were analyzed with Targetscan. A total of 6,379 mRNAs were predicted as the potential targets of 14 miRNAs. We obtained 5,839 target genes by deleting the duplicate and keeping the unique value (the data are not shown). Next, we identified the co-expressed mRNAs with the same expression trend both in the predicted mRNAs and the 1,342 differentially expressed genes in the above-mentioned five macrophage subset populations by manual scrutinizing. Briefly, if the expression of miRNAs was up-regulated (higher in the exosomes of CAD patients vs. healthy control), we thought that the predicted mRNAs were down-regulated in CAD patients. Then, we selected the same genes with the same expression trend (down-regulated) from 1,342 DEmRNAs in the five macrophage subset populations. A total of 38 DEmRNAs in five clusters were selected as the target genes of 14 DEmiRNAs (shown in Table 5).

**TABLE 5:** The DEmiRNAs/mRNAs in five monocyte-related populations.

**TREM2 high Mφ**	**C1qb**	**miR-15b-5p**	**miR-4498**	
	Anxa5	miR-1226-5p	miR-4498	
	Igf1	miR-1304-5p	miR-29c-5p	miR-433-3p
		miR-4498	miR-320c	miR-452-5p
	Ctsb	miR-221-5p	miR-29c-5p	miR-1226-5p
		miR-4498		
	Ms4a7	miR-452-5p	miR-4498	
	Syngr1	miR-200c-3p	miR-4498	
	Hexa	miR-320c		
	Ctss	miR-1226-5p	miR-320c	miR-4498
Mφ-DC	Syngr2	miR-4498	miR-200c-3p	
	Gm2a	miR-433-3p	miR-320c	miR-4498
	Cd74	miR-320c	miR-4498	
	Flt3	miR-433-3p	miR-1226-5p	miR-320c
	Olfm1	miR-320c	miR-4498	miR-200c-3p
	H2afz	let-7f-2-3p	miR-1304-5p	
	Lsp1	miR-4498		
	Plbd1	miR-542-3p		
	Rogdi	miR-433-3p		
Inflammatory Mφ	Cd83	miR-15b-5p	miR-29c-5p	
	Egr1	miR-1304-5p	miR-15b-3p	
	Socs3	miR-221-5p		
	Csf1r	miR-320c		
	Nfkbia	miR-1304-5p	miR-4498	
	C3ar1	miR-15b-5p	miR-1304-5p	
	Cd81	miR-433-3p	miR-4498	miR-1226-5p
	Marcksl1	miR-320c		
	Fcer1g	miR-196b-5p	miR-433-3p	
	Msrb1	miR-433-3p		
	F10	miR-4498		
Monocyte	Cstb	miR-1226-5p	miR-4498	
	Plin2	miR-1226-5p		
	Sat1	miR-1226-5p		
	Lst1	miR-1304-5p		
	Msr1	miR-433-3p	miR-320c	miR-4498
	Fcgrt	miR-1304-5p		
	Mrc1	miR-452-5p		
Resident Mφ	Serinc3	miR-1304-5p		
	Txnip	miR-1304-5p	miR-433-3p	miR-4498
	Cfh	miR-4498		

### Construction of an Interacted Network of RNAs and Macrophage Clusters

**FIGURE 4:**
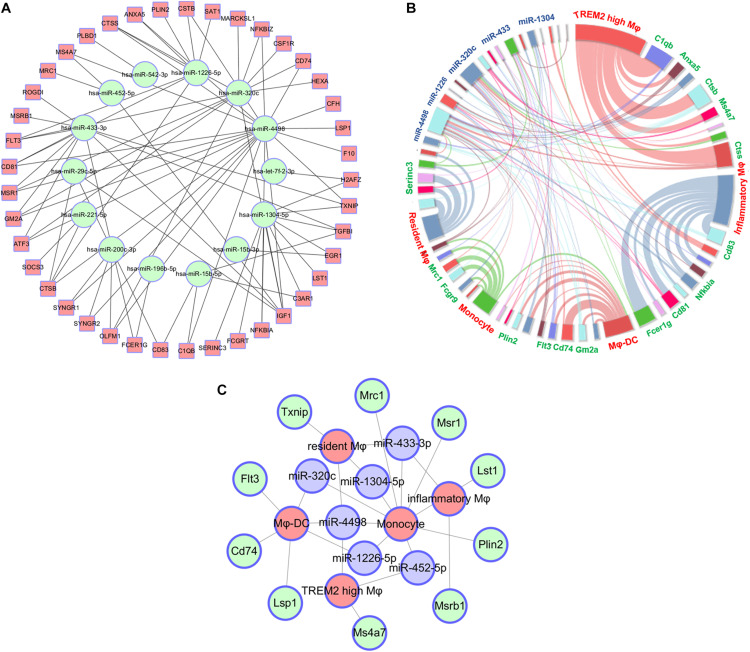
Construct of interacted network of RNAs and monocyte-related populations. **(A)** The co-expression networks based on the screening of miRNA–mRNA pairs. **(B)** Highchart showing the relationships between miRNAs, mRNA, and five monocyte-related clusters. **(C)** The interacted network constructed by the key six miRNAs, 10 mRNAs, and five monocyte-related populations.

Based on the screening of miRNA–mRNA pairs, the co-expression networks were drawn with Cytoscape 3.7 to find out the key RNAs (Figure 4A). Each point consists of multiple-weighted links to other points in the highchart to visualize the relationships among miRNAs, mRNAs, and five monocyte-related populations. The key nodes were selected by dependency wheel series (≥ 5) in this network (Figure 4B). Depending on the highchart, we constructed the interacted network of key nodes, including six miRNAs (miR-433-3p, miR-320c, miR-1304-5p, miR-1226-5p, miR-452-5p, and miR-4498), 10 mRNAs (Txnip, Mrc1, Msr1, Lst1, Flt3, Cd74, Lsp1, Ms4a7, Msrb1, and Plin2), and five clusters (Figure 4C).

### Validation of DERNAs by Real-Time qRT-PCR

**FIGURE 5:**
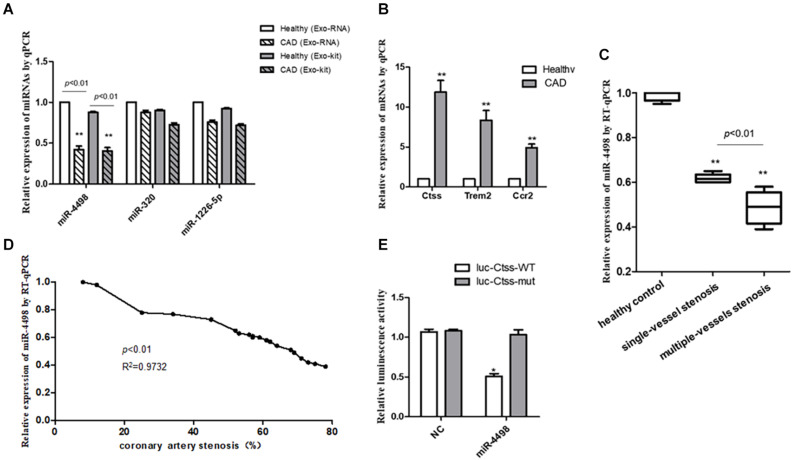
The quantitation of RNAs to validate the predicted results. **(A)** The quantitation of miRNAs isolated from plasma exosomes directly with exoRNeasy Midi Kits or miRNAs in exosomes isolated with exoEasy Max Kit as detected by RT-PCR. **p < 0.01 vs. healthy control, *n* = 3/group. **(B)** The quantitation of mRNAs in peripheral blood mononuclear cells was detected by RT-qPCR. **p < 0.01 vs. healthy control, *n* = 3/group. **(C)** The comparison of levels of mir-4498 between the patients with single-vessel stenosis (*n* = 6) and those with multiple-vessel stenosis (*n* = 9) vs. healthy controls (*n* = 5). **p < 0.01 vs. controls. **(D)** The correlation analysis between the levels of miR-4498 and the percentage of coronary artery stenosis in patients. *n* = 20. **(E)** The luciferase reporter assays in THP1 cells transfected with wild-type or mutant miR-4498 luciferase vector and control. *p < 0.05 vs. normal control, *n* = 3/group.

To reveal the exact regulation on proinflammatory cytokines secreted from PBMCs by miRNAs derived from exosomes, we validated the expression of the three down-regulated miRNAs in the plasma exosomes of CAD patients in the study of RNA-seq (*n* = 3/group). The results showed that only miR-4498 by RNA-seq was significantly lower in CAD patients than in healthy control (*p* < 0.01), and there were no significant differences of miR-320c and miR-1226-5p in the two groups (*p* > 0.05) (shown in Figure 5A). To compare whether the RNA profile isolated from exosome with exoEasy Max Kit for *in vitro* experiments was consistent with the RNAs isolated from plasma exosomes directly with exoRNeasy Midi kits, we compared the expression of three down-regulated miRNAs by RT-PCR. The results showed the similar expression of RNA profile (Figure 5A, *n* = 3/group).

Then, we detected the mRNA expression in PBMCs from CAD patients by real-time RT-PCR to validate the previous prediction about the mRNAs which were up-regulated by the DEmiRNAs derived from the plasma of CAD patients vs. healthy control (*n* = 3/group) in order to reveal the regulatory effect of exosome–miRNA on the expression of inflammatory cytokines in PBMCs. The results showed that the mRNA levels of Ctss, Trem2, and Ccr2 were significantly higher in CAD patients than those in healthy controls (*p* < 0.01), as shown in Figure 5B.

### Relationship Between miR-4498 Level and Coronary Artery Stenosis

In light of the angiography findings, the CAD patients were divided into single- or multiple-vessel stenosis groups. The levels of miR-4498 were significantly lower in multiple-vessel stenosis (*n* = 9) groups than those in single-vessel stenosis (*n* = 6) groups (*p* < 0.01), and the levels in both of these two groups were down-regulated compared with that in the healthy control (*n* = 5) group (*p* < 0.01), as shown in Figure 5C. In addition, we selected part of the individuals to perform a correlation analysis between the levels of miR-4498 and the percentage of coronary artery stenosis. The results showed that the levels of miR-4498 were negatively correlative to the percentage of coronary artery stenosis (*p* < 0.01, *R*^2^ = 0.9732, *n* = 20), shown in Figure 5D.

### Luciferase Reporter Assay

The results showed that the enforced expression of miR-4498 could dramatically reduce the luciferase activity of the wild-type Ctss luciferase vector (*p* < 0.05), without affecting the luciferase activity of the mutant one, but the enforced expression of miR-4498 could not reduce the luciferase activity of the wild-type Ctss luciferase vector (Figure 5E, *n* = 3/group).

### Characterization of Plasma Exosomes

**FIGURE 6:**
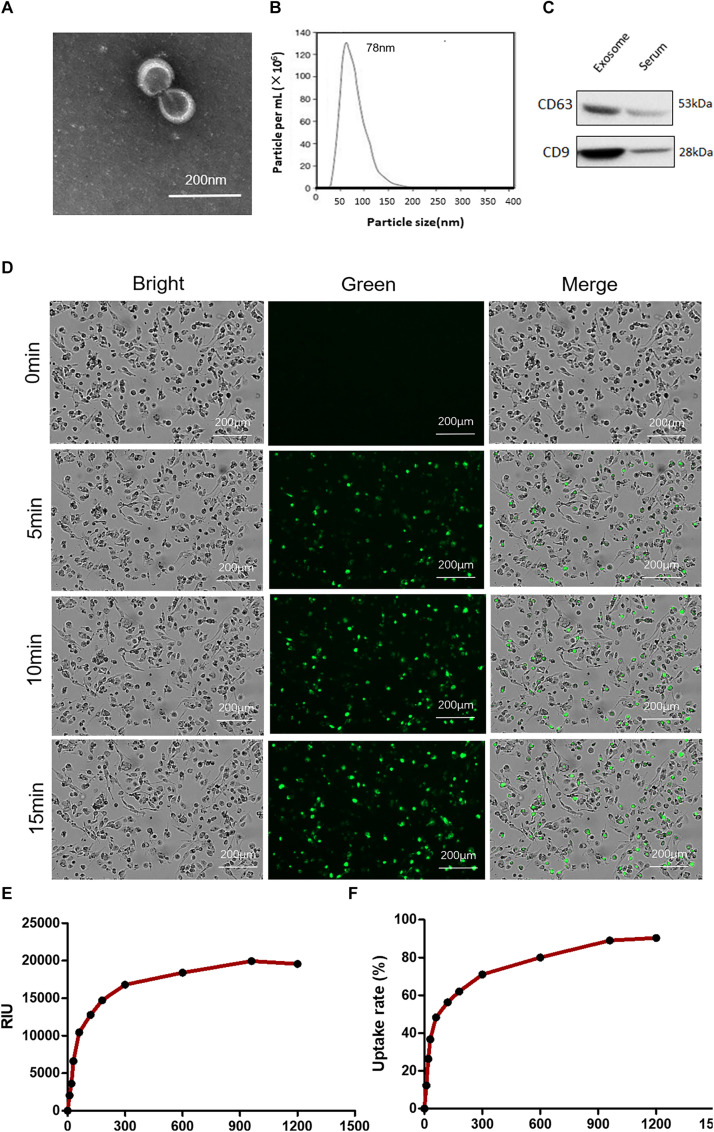
Characterization of plasma exosomes and uptake of exosomes by macrophages. **(A)** Electron microscopy observation of whole-mounted exosomes purified from plasma. **(B)** Average overall size distribution of exosomes from plasma using nanoparticle tracking analysis. **(C)** The proteins from isolated exosomes were initially characterized on Western blot to assess the expression of CD63 and CD9. **(D)** Internalization of PKH67 (green)-labeled exosomes in THP1 cells induced by phorbol-12-myristate-13-acetate was captured by living cell imaging. Fluorescent cellular imaging was carried out using the green fluorescent protein channel for PKH67 fluorescence-labeled exosomes (green). Cell counts were detected with bright field. **(E)** The relative object integral fluorescence was measured over time with the uptake of exosomes. **(F)** The percentage of uptake rate was calculated with the software.

The exosomes were characterized based on their morphology and size. The analyses by means of transmission electron microscopy confirmed the typical characterization of whole-mounted exosomes (Figure 6A). The particles of the exosomes were found in the range of 40–120 nm by NTA, supporting a multimodal size distribution of exosomes with a peak diameter of 70–120 nm (Figure 6B), consistent with previous reports (16). The protein content of exosomes isolated from plasma was characterized by Western blotting. The plasma exosomes from each patient were positive for the exosome markers CD63 and CD9 (Figure 6C).

### Internalization of PKH67-Labeled Exosomes

To address whether exosomes containing miRNAs can be internalized into THP1 cells, we first labeled the purified exosomes with the green fluorescent lipid dye PKH67 and incubated them with the cells. After incubation, green fluorescence-positive puncta were observed confluent in cultured cells (Figure 6D). The relative object integral fluorescence was measured over time with the uptake of exosomes, and the percentage of the uptake rate was calculated with the software (shown in Figures 6E,F).

### mRNA Expression in Macrophages After Uptake of Exosomes

**FIGURE 7:**
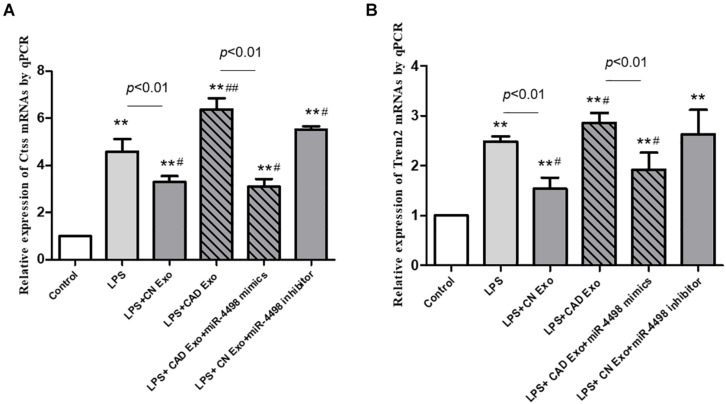
Gene expression of macrophages induced by exosomes from the plasma. The quantitation of mRNAs of Ctss **(A)** and Trem2 **(B)** in THP1 cells was detected by RT-qPCR. **p < 0.01 and *p < 0.05 vs. control, ^##^*p* < 0.01 and ^#^*p* < 0.05 vs. lipopolysaccharide; *n* = 3/group.

THP1 cells were differentiated into macrophages by PMA and cultured with lipopolysaccharide plus plasma exosomes to simulate an inflammatory microenvironment *in vivo* of CAD patients. Then, the cells were collected for RT-PCR assay to clarify the regulation of miR-4498 derived from exosomes. The results showed that the levels of Ctss and Trem2 mRNA were up-regulated as expected after having been induced by LPS plus exosomes from CAD patients (CAD exo) (*p* < 0.01 vs. control), and miR-4498 mimics down-regulated the levels of both mRNAs (*p* < 0.01 vs. LPS + CAD exo). While the levels of Ctss and Trem2 mRNA were down-regulated after being induced by LPS plus exosomes from healthy controls (CN exo) (*p* < 0.01 vs. LPS) and the miR-4498 inhibitor up-regulated the levels of both mRNAs (*p* < 0.01 vs. LPS + CN exo) (shown in Figure 7), we speculated that the miR-4498 derived from exosomes played a key role in regulating the mRNA levels of Ctss and Trem2.

## Discussion

Recent studies have shown that exosomes mediate the communication between cells and organs, which provides insights into the development of novel therapeutics for cardiovascular diseases. Researches demonstrate that the majority of miRNAs in plasma are encapsulated in exosomes (17). Exosomes carry these important cargo molecules throughout the body, sending signals to distant tissues and coordinating the system response (18). The miRNAs that they carry provide the cells with specific features that can reveal important insights into the origin of the cells and the pathogenesis mechanism in diseases (19). In cardiovascular diseases, such as acute myocardial infarction and atherosclerosis, the miRNAs, chemokines, cytokines, growth factors, and intracellular proteins in the microenvironment play an important role, many of which are enclosed with exosomes from multiple cells, such as cardiac cells, vascular endothelial cells, and immune cells (20–23).

Atherosclerosis is characterized by plaque formation and the infiltration of inflammatory foam cell macrophages (24, 25). In this research, we examined monocyte-related populations in plaque by single-cell transcriptomics and exosome miRNAs from CAD patients by RNA-seq to explore the internal regulatory mechanism. It has shown that exosomes participate in the cell crosstalk and play a critical role in the pathogenesis of CAD. Therefore, a further understanding of the biological functions surrounding exosomes in the context of immunity is necessary for the development of novel therapies.

The study has shown that five atherosclerosis-associated immune cell populations could be found in atherosclerotic aortas, including CD8 + T cells, monocyte-derived DCs, monocytes, and two populations of atherosclerosis-associated macrophages (TREM2^high^ macrophages and inflammatory macrophages) (6). Atherosclerosis-associated monocytes/macrophages showed high expressions of Adgre1, Cd14, Fcgr1, and Csf1r and a low expression of Ly6c2. The most significantly enriched genes in inflammatory macrophages included various proinflammatory chemokines (Cxcl2, Ccl3, and Ccl4), Tlr2, and Nlrp3. TREM2^hi^ macrophages displayed a unique gene signature with the expressions of Cd9, Hvcn1, and several cathepsins, except the most significantly enriched gene Trem2.

Ctss (cathepsin S), a member of the peptidase C1 family, is a lysosomal cysteine proteinase that participates in the degradation of antigenic proteins into peptides which are presented on MHC class II molecules (26, 27). When secreted from cells, this protein can remodel components of the extracellular matrix such as elastin, collagen, and fibronectin. This gene is involved in the pathology of many inflammatory and autoimmune diseases. The study has shown that the proteolytic activity of intracellular caspase 1 and extracellular Ctss in macrophages can be used as alternative biomarkers for lysosomal rupture and acute inflammation (28). Previous research demonstrated that Ctss was expressed by intimal macrophages as well as SMCs in human atherosclerotic arteries and involved in atherogenesis along with serine proteases and MMPs (29). The inhibition of Ctss could decrease the atherosclerotic lesions in ApoE^–/–^ mice (30).

The results confirmed our hypothesis that LPS could induce the differentiation of Trem2^high^ macrophages, while miR-4498 derived from plasma exosomes could inhibit the expression of inflammatory cytokines, such as Ctss and Trem2. In exosomes from CAD patients, the levels of miR-4498 were low, and these could not inhibit the expression of inflammatory cytokines, while the high levels of miR-4498 in exosome from healthy individuals could inhibit the expression of these inflammatory cytokines effectively and reverse the differentiation of monocytes/macrophages into anti-inflammatory phenotype.

This study suggests that tissue-derived exosomal miRNA might polarize monocytes (macrophages) into pro-inflammatory phenotype and thus further accelerate macrophage infiltration and chronic atherosclerotic inflammation. Circulating exosomal miRNAs potentially contribute to regulating molecular signaling networks in cardiovascular diseases. This study provides new insight regarding the pathogenic profile of exosomes in coronary artery disease. In future studies, we will further explore the pathogenesis of atherosclerosis based on more exosomal miRNAs regulating the function of macrophages.

## Data Availability Statement

The datasets presented in this study can be found in online repositories. The names of the repository/repositories and accession number(s) can be found in the article/supplementary material.

## Ethics Statement

The studies involving human participants were reviewed and approved by the institutional ethical committee of Liaoning University of Traditional Chinese Medicine. The patients/participants provided their written informed consent to participate in this study.

## Author Contributions

WC designed the majority of experiments. XL wrote the manuscript. XH and JW contributed to the data analysis of bioinformation. AZ helped in experimental design. DW and PC contributed to performed WB and gene assay. All authors contributed to the article and approved the submitted version.

## Conflict of Interest

The authors declare that the research was conducted in the absence of any commercial or financial relationships that could be construed as a potential conflict of interest.

## Footnotes

1 https://www.mdc-berlin.de/8551903/en/

2 http://www.mirbase.org/

3 http://www.targetscan.org

4 http://starbase.sysu.edu.cn

5 https://bioinfogp.cnb.csic.es/tools/venny/index.html

6 http://www.geneontology.org

7 https://jshare.com.cn/demos/JEtvoW

## Abbreviations

ApoE: apolipoprotein E
AS: atherosclerosis
CAD: coronary artery disease
CCL: C-C motif chemokine ligand
CXCL2: C-X -C motif chemokine ligand 2
FACS: fluorescence-activated cell sorting
GO: Gene Ontology
IL: interleukin
KEGG: Kyoto Encyclopedia of Genes and Genomes
LDLR: low-density lipoprotein receptor
LPS: lipopolysaccharide
miRNA: microRNA
MoDC: monocyte-derived dendritic cells
MRE: microRNA response element
M φ: macrophages
NLRP3: nucleotide-binding oligomerization domain leucine-rich repeat and pyrin domain containing 3
NTA: nanoparticle tracking analysis
PMA: phorbol-12-myristate-13-acetate
RNA-seq: RNA sequencing
RT-PCR: reverse transcription-polymerase chain reaction
TLR2: toll-like receptor 2
TREM2: triggering receptor expressed on myeloid cells.
